# Assessment of Response to Immunotherapy in Patients with Hodgkin Lymphoma: Towards Quantifying Changes in Tumor Burden Using FDG-PET/CT

**DOI:** 10.3390/jcm12103498

**Published:** 2023-05-16

**Authors:** Francesca Tutino, Elisabetta Giovannini, Silvia Chiola, Giampiero Giovacchini, Andrea Ciarmiello

**Affiliations:** 1 Nuclear Medicine Unit, Ospedale Civile Sant’Andrea, Via Vittorio Veneto 170, 19124 La Spezia, Italy; 2 Nuclear Medicine Unit, IRCCS Ospedale Policlinico San Martino, 16132 Genoa, Italy

**Keywords:** immunotherapy, Hodgkin lymphoma, FDG-PET, pseudoprogression, LYRIC, MTV

## Abstract

Immune checkpoint inhibitors are currently the standard of care for many advanced solid tumors, and they have been recently approved for the treatment of relapsed/refractory Hodgkin lymphoma and primary mediastinal B cell lymphoma. Assessments of the response to immunotherapy may be complicated by the occurrence of the flare/pseudoprogression phenomenon, consisting of initial tumor enlargement and even the appearance of new lesions, followed by a response, which may initially be indistinguishable from true progression. There have been efforts to characterize and capture the new patterns of response observed during immunotherapy, namely, pseudoprogression and delayed response, and several immune-related response criteria have been proposed. Confirming progression on a subsequent scan and measuring the total tumor burden are both common in immune-related criteria. Due to the peculiarity of hematologic malignancies, lymphoma-specific immune-related criteria have been developed (LYRIC), and they have been evaluated in research studies in comparison to the Lugano Classification. In this review work, we illustrate the evolution of the response criteria in lymphomas from the first CT-based criteria to the development of the PET-based Lugano Classification, further refined to take into account the flare phenomenon encountered during immunotherapy. We also describe the additional contribution of PET-derived volumetric parameters to the interpretation of responses during immunotherapy.

## 1. Introduction

In the last decade, novel biological agents with an immune mechanism have entered the clinical world; the newest agents are immune checkpoint inhibitors. Nowadays, immune checkpoint inhibitors represent the standard of care for advanced melanoma, non-small-cell lung cancer, renal carcinoma and head and neck tumors [1,2,3]. In the last decade, the impressive results of phase I and II studies exploring the effectiveness and safety of PD-1 inhibitors in Hodgkin lymphoma (HL) [4,5] and primary mediastinal B cell lymphoma (PMBCL) [6] granted the accelerated approval of anti-PD-1 by the FDA without a confirmatory phase III study. In 2016, nivolumab was approved by the FDA for the treatment of relapsed/refractory classical HL (cHL) after autologous stem cell transplantation and brentuximab vedotin as the first hematologic indication. Pembrolizumab was approved for relapsed/refractory cHL after at least three lines of therapy in 2017 and for relapsed PMBCL after the failure of two or more lines of therapy in 2018 (Keynote 013 study).

The impact of immune checkpoint inhibitors on the treatment of HL is related to the unique property of HL of being constituted only by a minority of malignant cells (Reed–Stemberg cells) embedded in an abundant microenvironment, whose cells overexpress PD1-PDL1 due to a genetic aberration in the 9p23-24 locus. Immune checkpoint inhibitors are of minor importance in non-Hodgkin lymphoma (NHL); no immune checkpoint inhibitor approval exists for NHL. However, for relapsed/refractory NHL, the option of chimeric antigen receptor T (CAR-T) cell therapy is gaining ground. CAR-T therapy was recently approved by the FDA and EMA for the treatment of relapsed/refractory diffuse large B cell lymphoma.

Immune checkpoint inhibitors, working with an immune mechanism, may cause a transient increase in tumor burden due to inflammation, named pseudoprogression, and they may alter tumor metabolism, yielding false positive and false negative results on FDG-PET/TC. In recent years, novel response criteria were designed in an attempt to capture these additional response patterns beyond those observed in conventional chemotherapy.

In this review work, we examine the evolution of response criteria from the first efforts to describe the effects of conventional chemotherapy on tumor growth to the development of lymphoma-specific criteria and their refinement to be suitable to capture the benefit provided by immunotherapy. We also describe the contribution of additional FDG-PET/CT quantitative parameters, such as metabolic tumor volume (MTV) and total lesion glycolysis (TLG), to assessing changes in tumor burden in the course of immunotherapy.

## 2. Immunobiology of Immune Checkpoints

Tumor cell growth is promoted by the ability of tumor cells to “escape” from the immune system and to be immunotolerant. Tumor cells lose their immunogenic antigens and manipulate the microenvironment dysregulating immune checkpoints to express inhibitory signals [7,8,9]. The rationale of immunotherapy is to restore a florid T-cell cytotoxic response directed against the tumor, and this can be achieved either by activating stimulatory checkpoints or by inhibiting inhibitory checkpoints [10].

The most relevant inhibitory checkpoints are programmed death cell receptor 1 (PD1) and cytotoxic T-lymphocyte-associated protein 4 (CTLA-4), both being receptors expressed on the T-cell surface inducing T-cell anergy. PD1, through the interaction with its ligand, programmed death cell ligand 1 (PDL1), expressed in antigen-presenting cells (APCs), activated T cells and tumor cells, inhibits the T-cell cytotoxic response [11]. CTLA-4 inhibits T-cell proliferation by blocking the costimulatory molecules of the B7-CD28 superfamily expressed on APCs [12].

The knowledge about the expression of immune inhibitory checkpoints in hematologic malignancies has been illustrated in a recent review work by Witkowska and Smolewsky [13]. HL widely overexpresses PD1/PDL1 due to a widespread genetic alteration in the locus 9p23-24 and the subsequent activation of Janus kinase 2 [14]. PMBCL shows a high expression of PD1 ligands, especially the EBV-positive subtype, probably mediated by virus latent proteins [15,16]. Follicular lymphoma (FL), originating from B germinal centers similarly to HL and PMBCL, may express PD1 ligands [17]. CTLA-4 expression, of which little is known about, might be observed in T-cell lymphomas and Sezary syndrome [18].

## 3. Review of PET-Based Criteria for Response Assessment

### 3.1. Background and Assessment of Response to Conventional Chemotherapy

The first effort towards the standardization of assessments of the response to cancer treatment was a handbook published in 1979 promoted by the World Health Organization (WHO) [19]. The WHO criteria stated the concept of the tumor bidimensional measurement of tumor burden as a sum of the products of lesion diameters before and after therapy and established the four response categories still currently in use: complete response, partial response, stable disease and progressive disease.

The first guidelines to incorporate the metabolic data provided by FDG-PET/CT in response assessment were the European Organization for Research and Treatment of Cancer (EORTC) criteria, released in 1999 [20]. The reference region for complete metabolic response was the background adjacent to lesions. The main goal of the EORTC criteria was to evaluate the viability of residual masses: based on metabolic activity, it was feasible to discriminate fibrotic/necrotic changes from residual tumors.

A new set of joint EORTC/National Cancer Institute CT-based guidelines for response assessment, the Response Evaluation Criteria in Solid Tumors (RECIST), was first published in 2000 [21] and then revised and updated in 2009 (RECIST 1.1) [22]. In contrast to the bidimensional assessment of the WHO criteria, being laborious and time consuming, the RECIST criteria rely on a unidimensional assessment of the largest axial diameters of the tumors [23]. Moreover, RECIST introduced the concept of target lesions. 

In the same year, 2009, on the heels of RECIST, Wahl et al. published the PET Response Criteria in Solid Tumors (PERCIST) [24]. Similarly to RECIST, the PERCIST criteria rely on the assessment of residual metabolic activity in target lesions (hottest lesions). The remarkable innovations of PERCIST are the introduction of SUV lean (SUL, SUV normalized for lean body mass) and SUL peak and the definition of the minimum measurable activity as 1.5 times hepatic activity.

Due to the peculiarity of hematologic malignancies, specialized criteria for response assessment in lymphomas were developed. The first effort to design response criteria specific for lymphomas was the International Working Group (IWG) criteria [25], sponsored by the National Institute of Health, published in 1998. The IWG criteria were CT-based criteria, and they introduced a fifth response category, namely, complete response/unconfirmed (CRu), defined as the persistence of residual nodal masses despite a reduction greater than 75% in the sum of the product of diameters. CRu reflects the difficulty of assessing the origin of residual masses based purely on radiological data. 

In the early 2000s as the fast growth of PET began and as PET/CT tomographs were developed, the gain in accuracy provided by PET, able to assess the viability of residual masses, was recognized, leading to the proposal of the so-called IWG+PET criteria by Juweid et al. in 2005 [26]. Soon after in 2007, in the context of a project promoted by the German Study Group, the International Harmonization Project, two publications by Cheson et al. [27] and by Juweid et al. [28] updated the IWG criteria, embodying PET in the response evaluation. These modified criteria were based on an integrated evaluation of CT and PET. The PET evaluation was qualitative and provided a positive or negative classification based on a comparison of activity in residuals with activity in reference regions (mediastinal blood pool for residual masses greater than 2 cm and adjacent background for smaller lesions). The assessment of viability in residual tumors enabled by PET/TC led to the elimination of the ambiguous CRu category.

In 2009, an International workshop held in Deauville (France) formulated novel response criteria, the Deauville Score (DS) [29,30]. DS is a five-point scale based on a visual comparison of activity in residual tumors with activity in reference regions (mediastinal blood pool and liver). In 2013, at the 12th International Conference on Malignant Lymphomas, the Lugano Classification was developed [31], a body of consensus recommendations for staging and response assessment in lymphomas. According to the Lugano guidelines, both contrast CT and PET have to be performed in the setting of response assessment. Separate sets of response criteria for CT and PET evaluations were published. For PET interpretation, DS was adopted. DS, being simple and easy to implement, had widespread diffusion and underwent a process of standardization across centers, becoming the gold standard for response assessment in lymphomas. 

In the case of uncertainty of DS attribution, research groups active in the field recommend confirming visual evaluations with the SUV ratio between residual tumors and reference regions [32]. Recently, quantitative extensions of DS were also developed, particularly qPET [33,34], but these methods have not yet been prospectively validated and need standardization.

**Figure 1:**
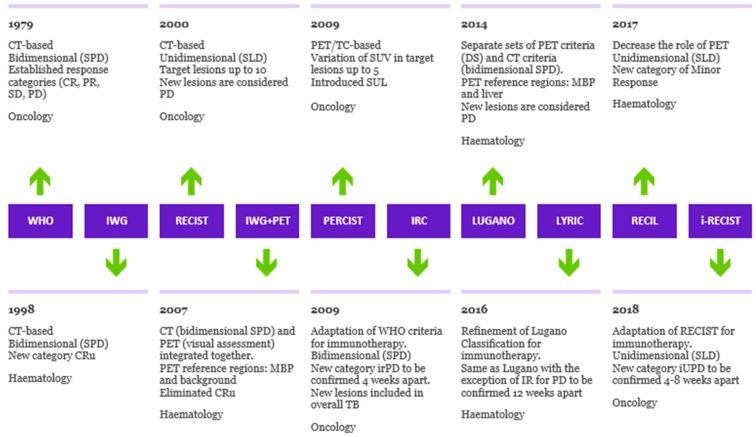
Evolution of criteria for response to cancer treatment. Timeline illustrating the evolution of response criteria over time in oncology and hematology, outlining the differences in method of tumor measurement, PET interpretation and assessment of progression of disease. SPD: sum of products of diameters. SLD: sum of longest axial diameters. MBP: mediastinal blood pool. SUL: standardized uptake lean mass. DS: Deauville Score. Cru: unconfirmed complete response. irPD: immune-related progression of disease. IR: indeterminate response. iUPD: immune-unconfirmed progression of disease.

The evolution of the response criteria in oncology and hematology over time is presented in Figure 1.

### 3.2. Pseudoprogression and Hyperprogression

The Lugano Classification was designed to assess the response to traditional chemotherapy or conventional chemo-immunotherapeutic regimens, including rituximab. The patterns of response to immunotherapy differ from the patterns observed in conventional treatments. Usually, response occurs early after immunotherapy, and, consequently, an early response evaluation after two–three cycles of therapy is advisable. Response assessment may be confounded by the phenomena of delayed response and flare/pseudoprogression. Delayed response consists of a late objective response in the course of treatment, after initial tumor growth and apparent progression of the disease. Flare/pseudoprogression was first described in lymphomas and chronic lymphocytic leukemia receiving lenalinomide as a rapid increase in the size of lymph nodes, often painful, accompanied by fever and lymphocytosis [35,36,37]. Flare/pseudoprogression is defined as an increase in the size of baseline lesions and even the appearance of new lesions when the patient is clinically improving. It represents an apparent progression on imaging, in the absence of clinical deterioration of the patient, and it is followed by a response. Pseudoprogression usually occurs early during treatment. The increase in the size of baseline lesions is an inflammatory phenomenon due to T-cell recruitment, NK activation and a massive release of cytokines [38]. It is crucial to recognize pseudoprogression and to not discontinue treatment before achieving clinical benefit.

Hyperprogression, defined as a rapid acceleration of tumor growth, is a new aggressive pattern reported in a fraction of lung cancer, melanoma, renal carcinoma [39] and head and neck carcinoma [40] cases treated with anti-PD-1/PD-L1. Compared to pseudoprogression described above, hyperprogression is a disruptive phenomenon, and it is not prone to uncertainty in interpretation.

### 3.3. Assessment of Response to Immunotherapy

Atypical responses encountered in patients under immune checkpoint blockade, due to delayed responses and pseudoprogression, and additional response patterns beyond those of conventional chemotherapy classified by the WHO and RECIST criteria were shown to be associated with survival benefit comparable to typical responses [41] and needed to be taken into account in response assessment. There have been efforts to characterize these phenomena and to incorporate them into novel response criteria.

In 2009, a publication by Wholchok et al. proposed the Immune-Related Response Criteria (IRC) [41], novel CT-based immune therapy response criteria adapted from the WHO criteria, based on the experience of community workshops using data from patients with advanced melanoma treated with ipilimumab. Across this cohort of patients, four patterns of response to ipilimumab were reported. Two patterns were captured by conventional response criteria: (1) a shrinkage in baseline lesions without new lesions and (2) “stable” disease, eventually followed by a slow steady decline of tumor burden (TB). The other two were new and were beyond conventional response assessment: (3) response after an initial increase in TB and (4) a reduction in overall TB concomitantly with the appearance of new lesions. 

The main statements of the IRC can be resumed as follows:

1. Immune-related progression (irPD) of disease needs to be confirmed in a subsequent scan at least 4 weeks later in the absence of clinical deterioration and the worsening of laboratory parameters, aimed to uncover delayed response and pseudoprogression phenomena. PD confirmation is required before withdrawing treatment.
2. Stable disease is considered a therapeutic effect and a surrogate end-point for clinical benefit in contrast to assessment of the response to conventional cytotoxic therapy.
3. Overall, TB has to be measured, even when new lesions appear. The threshold for SD and partial response (PR) are the same as that in the WHO criteria, but new lesions are included in TB assessment.

The IRC have been implemented into clinical trials evaluating immune checkpoint inhibitors in solid tumors.

In 2013, the IRC were adapted to the unidimensional RECIST criteria and called Immune-Related RECIST (irRECIST) [42]. In 2017, the RECIST working group adapted the RECIST 1.1 criteria to the new body of knowledge about the patterns of response to immunotherapy in solid tumors and developed the so-called Immune-RECIST (i-RECIST) [43]. i-RECIST have a new response category of “immune unconfirmed progression” that requires confirmation on a subsequent scan within 6–8 weeks, accounting for the occurrence of pseudoprogression and delayed response. 

In the studies on the immune checkpoint blockade in LH and N, a similar incidence of delayed response and flare/pseudoprogression, and response patterns similar to those reported in solid tumors have been observed. However, merely translating the IRC in the setting of response assessment in lymphomas was not considered totally appropriate for the following reasons: First, over time, there was an independent evolution of the response criteria for solid tumors and lymphomas. Response in solid tumors is assessed using morphologic unidimensional criteria, the RECIST criteria, whereas response in lymphomas is evaluated using the Lugano Classification based on PET/TC and on a bidimensional assessment of lymph node size on CT. Second, progression is defined by the WHO criteria as an increase in size >25% of the sum of the product of the diameters of solid tumors, whereas in lymphomas, an increase in the size of a single lymph node accompanied by PET positivity is adequate to discern progression. Third, response assessment in solid tumors is based on a dimensional evaluation of masses, always considered abnormal, whereas in the setting of lymphomas, residual masses do not have just an interpretation, since they can represent fibrotic/necrotic changes, according to metabolic activity.

**Figure 2:**
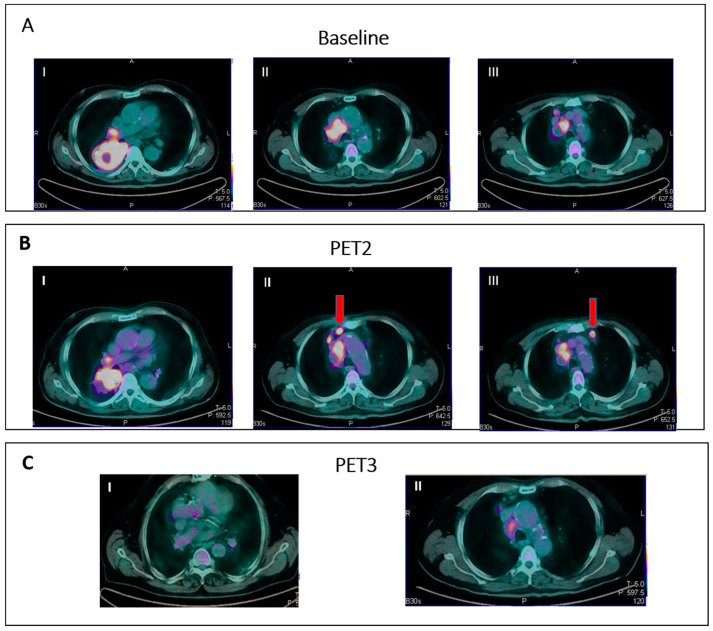
IR (2): Pseudoprogression in a patient on nivolumab for Hodgkin lymphoma. Panel (**A**) shows baseline disease. Panel (**B**) (II–III) shows the appearance of new nodal lesions (red arrows) in early PET evaluation after four cycles of immunotherapy. PET/TC evaluation at a later time point (**C**) demonstrates regression of the nodal flares and metabolic response.

**Figure 3:**
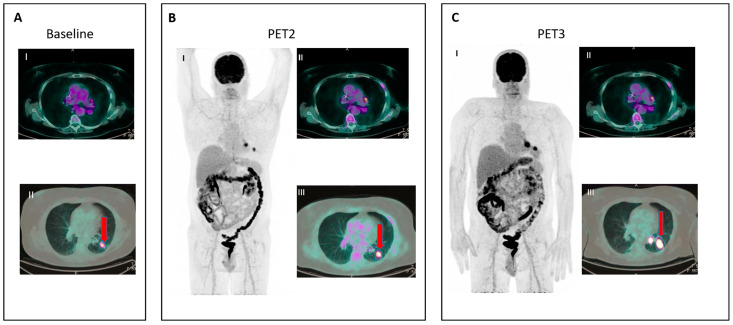
IR (3): Panel (**A**) shows baseline lesions (red arrow). Early PET evaluation (**B**) during nivolumab for Hodgkin lymphoma shows increase in FDG uptake (red arrow) in baseline lesions without concomitant increase in size. At subsequent PET evaluation (**C**), there is a concordant increment in size (red arrow), and criteria for true progression are met.

To address these issues, in 2016, the LYRIC criteria (Lymphoma Response to Immunomodulatory Therapy Criteria) [44] were developed as a refinement of the Lugano Classification accounting for features specific of immunotherapy. In the LYRIC criteria, a CT-based size assessment and a PET/TC evaluation are integrated together. LYRIC introduced the novel category of indeterminate response (IR) to account for flare/pseudoprogression and delayed response, requiring a confirmatory study, either a biopsy or subsequent imaging within 12 weeks. Three types of IR were identified:

- IR(1): Progression (defined as >50% increase in overall TB) in the first 12 weeks of therapy without clinical deterioration.
- IR(2): Appearance of new lesions in the context of overall TB stability (Figure 2).

- IR(3): Increase in the uptake of existing lesions without a concomitant increase in lesion size and number (Figure 3).

The LYRIC criteria were applied in studies assessing the response to immunotherapy in lymphomas and were compared with the Lugano Classification.

In 2017, with the aim of unifying the response criteria in lymphoma with the response criteria in solid tumors in the context of clinical trials evaluating new therapeutic agents in a mixed population of patients with lymphoma and patients with solid tumors, an international working group developed the Response Evaluation Criteria in Lymphoma (RECIL) [45]. RECIL looks at the RECIST criteria, proposing a unidimensional evaluation of the sum of the longest axial diameters in a maximum of three target lesions, instead of the sum of the product of diameters in up to six target lesions as suggested by the Lugano criteria. Based on the hypothesis that new therapeutic agents can alter a tumor’s metabolism and, thus, have the potential to increase false-positive and false-negative FDG-PET results, RECIL decreased the role of PET in response assessment in lymphomas. Although in the Lugano Classification, complete response (CR) was represented by PET negativity (DS 1–3) regardless of lesion size, in RECIL, the CR response category requires a shrinkage >30% of lesions besides PET negativity. The PR category was also modified to capture the mixed responses encountered with novel treatments. In the Lugano Classification, the increase in size >50% of a single lesion is sufficient to discern PD, even if other lesions concomitantly decrease in size. In contrast, in RECIL, similarly to the IRC and LYRIC seen above, the overall tumor burden is considered, and this case may discern PR, defined as a decrease in size >30% of overall TB accompanied by PET positivity (DS 4 or 5). RECIL introduced a novel provisional category of minor response, defined as a shrinkage of lesions >10% and <29% accompanied by any PET status, aiming to account for a response that does not fulfill the criteria for traditional response categories but may be associated with survival benefit. A comparison of the Lugano Classification, LYRIC and RECIL 2017 is presented in Table 1.

**Figure 4:**
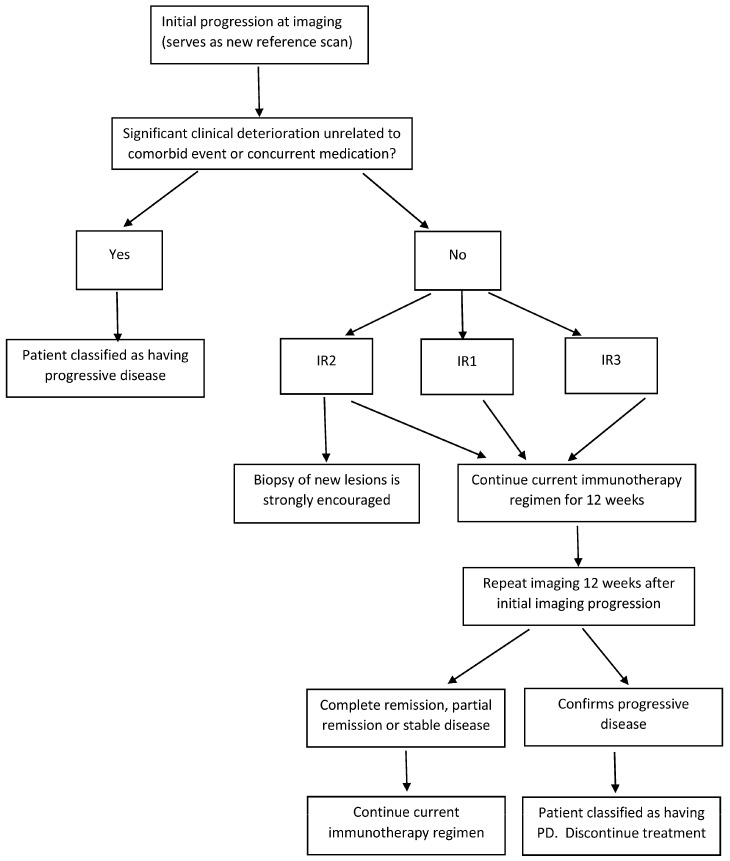
Flowchart of assessment of response to immunotherapy in lymphoma.

For an assessment of the response to immunotherapy in lymphomas, FDG-PET should be performed at baseline and repeated after three–four cycles (at 9–12 weeks). Immune checkpoint inhibitors induce inflammation that can translate into increased FDG uptake and even into the appearance of new lesions in the absence of true progression. In the assessment of patients with lymphoma during the course of immunotherapy, collaboration between clinicians, radiologists and PET readers in the context of a multidisciplinary approach is advisable in equivocal and challenging cases to discriminate treatment-induced inflammation/pseudoprogression from true progression. Decisions must be based on a repeated scan taken 12 weeks later. A re-biopsy, when feasible, might be necessary in cases of persistent FDG uptake, and it is encouraged in cases with the appearance of new lesions of indeterminate origin. We illustrate a possible algorithmic approach to patients with HL on immunotherapy in Figure 4.

## 4. Contribution of PET/CT-Derived Volumetric Parameters to Response Assessment

The morphologic CT-based criteria most widely adopted, the RECIST criteria, rely on a unidimensional assessment of target lesions, with up to five (two per organ maximum) intended to represent a sample of the total TB. Indeed, the assessment of the entire TB using CT in an individual patient is time consuming and complex. In contrast, by using PET/CT, exploiting the quantitative potential of parametric images, foci that accumulate FDG can be outlined by grouping together pixels with SUV above a chosen threshold (typically 41% of the maximum), quantified and summed up, with the aid of semiautomatic software for segmentation and with minimal manual intervention [46]. It is feasible to measure the total TB as metabolically active volume (MTV). Consequently, it is possible to easily assess the variations in TB between baseline and after therapy. Total lesion glycolysis, defined as MTV multiplied by SUVmean, can also be assessed using semiautomatic software, combining volumetric data with metabolic parameters. 

EANM guidelines for the use of PET/CT to evaluate the response to immunotherapy recommend performing the computation of volumetric parameters at baseline to study their modifications later during treatment [47].

The additional contribution of PET-derived volumetric parameters has been evaluated in recent research studies (Table 2). Two single-center, retrospective studies [48,49] suggested that the SUV metric is suitable to evaluate the response to immunotherapy in relapsed/refractory HL. They outlined a significantly greater MTV (ΔMTV) and TLG reduction (ΔTLG) in responders (CR and PR according to DS) than in non-responders. A study by Castello [49] and colleagues also showed that, in the majority of responders (29/31), tumor burden shrinkage was greater than 50%. In this study, the variation in the tumor burden metrics at an early evaluation (8 weeks) correlated with variation at later time points and accurately predicted the long-term outcomes of the patients.

The feasibility of response assessment using PET-derived volumetric parameters has been evaluated in small cohorts of naïve patients receiving HL therapy. A study by Savas et al. [50] in 13 patients with newly diagnosed HL assessed ΔMTV and ΔTLG after 3 sequential cycles of pembrolizumab. Based on the analysis of the response rates at the end of treatment, this study suggested that the response to pembrolizumab is better captured by the dramatic decline in TB at an early assessment compared to conventional criteria, namely, DS and SPD. Similarly, a study by Voltin [51] in 53 patients with early unfavorable HL (stage II) treated with nivolumab showed an early near-complete MTV reduction (ΔMTV 91%), despite there being lower rates of CR assessed with conventional criteria. Based on the outcome analysis on a follow-up period of 12 months, the authors suggested that conventional criteria could underestimate the response in this cohort of stage II HL. 

Recently, considering tumor heterogeneity and differences in therapy response, artificial intelligence (AI) approaches, radiomics and machine learning algorithms, have emerged as non-invasive technologies using medical imaging analyses. AI can extract significant quantitative data from patients’ medical images and correlate image features with diagnostic and therapeutic outcomes [52,53]. Radiomics has been applied in lymphomas to examine baseline FDG-PET for differential diagnosis from other malignancies and in evaluations of bone marrow involvement and pre-treatment risk [54,55], but, currently, no data are available for radiomic analyses in the context of assessments of the response to immunotherapy in lymphomas [56,57].

As the state of the art, it is recommended to assess MTV and TLG before treatment and during treatment in scheduled PET scans to quantify changes in tumor burden, as this can orient the interpretation of the response to immunotherapy.

## 5. Immune-Related Adverse Events

Immune-related adverse events (irAEs) represent a major cause of treatment discontinuation and a confounding factor in response assessment at imaging. However, the side effects experienced during immunotherapy seem to be better tolerated than the side effects due to cytotoxic therapy; a lower risk of serious (grade III–IV) adverse events has been reported [58].

IrAEs are related to the recruitment of an immune infiltrate in the organs involved. From this perspective, IrAEs can be seen as an “undesirable” sign of immune activation or rather that immunotherapy is acting effectively, as suggested by some studies reporting an association between irAEs and treatment efficacy [59,60].

The first sign of immune activation is the inversion of the liver-to-spleen ratio, which may be accompanied by splenomegaly. Speen activation can be associated with a mild diffuse uptake in bone marrow. In an early phase, reactive lymph nodes may be seen in the basin of the tumor. A sarcoid-like reaction, consisting of an increased uptake in mediastinal hilar lymph nodes and pulmonary granulomatosis, can also occur. These phenomena are transient, self-limiting and require monitoring until their resolution.

**Figure 5:**
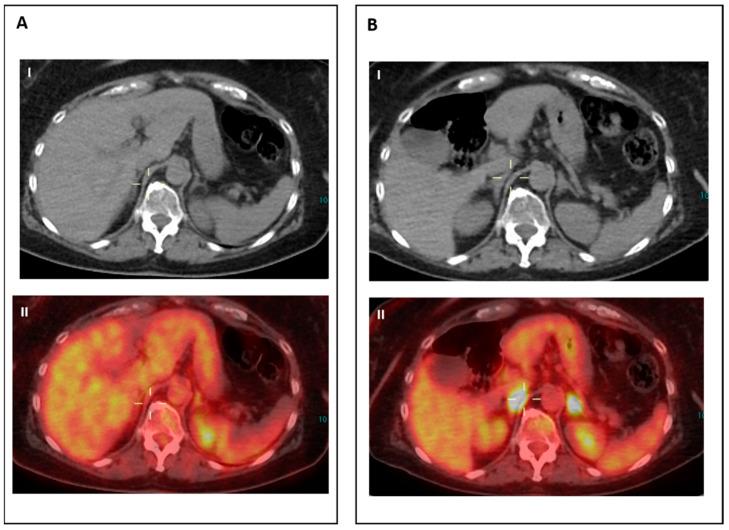
Adrenalitis: patient with advanced melanoma. Panel (**A**) shows CT (I) and PET/CT (II) during therapy with tyrosine kinase inhibitors; adrenal glands appear normal. Note the appearance of intense hypermetabolism in the adrenal glands (box (**B**), II) accompanied by symmetrical enlargement in CT images (box (**B**), I) during combined nivolumab plus ipilimumab treatment.

**Figure 6:**
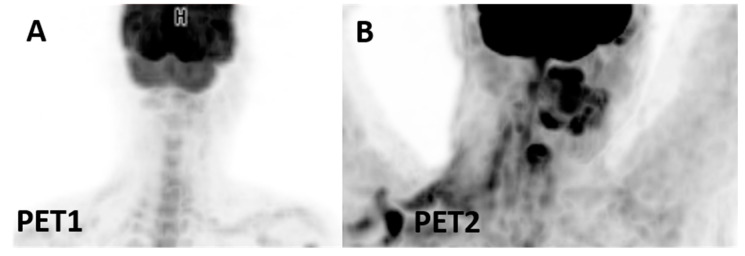
Nivolumab-induced thyroiditis: (**A**) no uptake in the thyroid gland at baseline. (**B**) appearance of diffuse mild uptake in the thyroid gland in a patient receiving nivolumab for Hodgkin lymphoma who developed thyroiditis.

IrAEs can affect any organ, particularly the endocrine system’s glands (hypophysitis, thyroiditis and adrenalitis). A symmetrical enlargement of the adrenal gland with diffusely increased FDG uptake can be due to adrenalitis (Figure 5). With particular reference to nivolumab-induced thyrotoxicity, in a recent metanalysis by Barroso-Sousa and colleagues [61], incidence rates of 6.5% for hypothyroidism and of 2.5% for thyrotoxicosis are reported. Nivolumab-induced thyroid dysfunction is due to painless thyroiditis, characterized in most cases by an early-onset, transient, thyrotoxic phase, commonly followed by hypothyroidism. A research study investigated the underlying mechanism of the thyroid [62], suggesting that, since normal thyroid tissue expresses PDL1 and PDL2 mRNA and proteins, PD1 pathway blockade impairs immunotolerance and can induce autoimmune thyroiditis in the absence of TRAb positivity. The appearance of a diffusely increased FDG uptake in the thyroid gland in the course of immunotherapy (Figure 6) should be considered suggestive of incipient thyroiditis, even before clinical manifestation and obtaining laboratory findings [63].

A threatening irAE that can limit treatment cycles is checkpoint-inhibitor-related pneumonitis (CPI). An accurate diagnosis of CIP can be difficult since, during the treatment of oncological patients, other factors (infections, radiation therapy and other drugs) are often mixed. In recent years, several studies [64,65] have demonstrated the potential of CT radiomics to differentiate CIP from other conditions, such as radiation-induced pneumonitis, leading to the development of the Rad-score [66], a robust model combining 11 imaging histological features with bilateral involvement and sharp borders.

The spleen must be critically checked: spleen enlargement and the inversion of the physiological spleen-to-liver ratio can occur in the course of immunotherapy, similarly to what is observed during conventional rituximab-based regimens, and should not be mistaken for lymphoma involvement.

IrAEs can be asymptomatic; in this context, FDG-PET/CT, being sensitive to foci of active inflammation, including those due to immune activation, has the unique ability to detect irAEs before clinical manifestation.

## 6. Future Perspectives

The selection of patients suitable to receive immunotherapy relies on several biomarkers (PD-L1 immunohistochemistry, immunohistochemistry for mismatch repair proteins, PCR-based assays for microsatellite instability and sequencing for tumor mutational burden on biopsy); however, they are not perfect and cannot accurately predict patient outcome. Furthermore, the current approach to assessing PD1 status cannot detect heterogeneity over time and across lesions due to the dedifferentiation of tumor clones, which may occur during therapy. To address these limitations, novel PET tracers, designed as antibodies or fragments of antibodies for specific immunotargets (immune-PET), were developed in the past few years. Immuno-PET offers the possibility to non-invasively image in vivo the whole body biodistribution of immune checkpoints and hold the potential to guide treatment decision making.

The first human PD-L1 PET study was conducted with ^89^Zr -atezolizumab in 22 patients with metastatic non-small-cell lung cancer (NSCLC), bladder cancer and triple-negative breast cancer [67]. The patients were imaged before starting immunotherapy. The study demonstrated a high tracer uptake in normal lymphoid tissue and sites of inflammation. Tumor lesions showed a generally high tumor uptake, with great intra-patient and inter-patient heterogeneity. [89Zr]Zr-atezolizumab uptake in tumor lesions correlated with the response to therapy, PFS and OS and outperformed immunohistochemistry on a fresh biopsy in the prediction of clinical response.

CD8 cells play an essential role in the cytotoxic response to tumors boosted by immunotherapy. The results of a clinical study evaluating the CD8 PET tracer 89ZED88082A, a zirconium-89-labeled one-armed antibody in solid tumors, have recently been published [68]. In this study, PET imaging was performed before and during immunotherapy. The pre-treatment biodistribution of the tracer showed specific CD8 targeting, with a high tracer uptake in normal lymphoid tissue. The tracer uptake in tumor lesions was variable within and between patients. The tumor uptake was higher in patients with mismatch-repair-deficient tumors and was correlated with CD8 cell density in tumors stained immunohistochemically. A higher SUVmax in tumor lesions at baseline showed a trend with improved OS. The results of serial 89ZED88082A imaging during immunotherapy showed a great spatial and temporal heterogeneity of the behavior of lesions in responders, providing an insight into the complex dynamic tumor microenvironment. A phase II trial investigating the efficacy of atezolizumab consolidation therapy in high-risk DLBCL is ongoing (HOVON 151) (NCT03850028) (https://clinicaltrials.gov last update posted 18 June 2019, accessed on 25 April 2023). In this study, patients are evaluated with sequential ^89^Zr-atezolizumab imaging before and after R-CHOP induction therapy, during atezolizumab consolidation therapy and at the time of suspected relapse. The HOVON 151 trial reflects the application of immune-PET in the clinical setting and may widen therapeutic options in lymphomas.

In the future, the promising field of immuno-PET may hopefully improve patient selection for immunotherapy and response assessment, and it may guide the development of new agents.

## 7. Conclusions

The criteria for response assessment in lymphoma have deeply evolved in the last decade, assigning an outstanding role to FDG-PET/CT. This path starts from the first lymphoma-specific CT-based criteria and leads towards the PET-based Lugano Classification, which, nowadays, represents the gold standard. The LYRIC criteria, the recent refinement of the Lugano Classification, were conceived to capture the new patterns of response observed during treatment with novel immunotherapy agents that have entered the clinic. In this context, PET/CT has the unique ability to assess response and uncover immune-related adverse events. PET/CT quantitative parameters, such as MTV and TLG, assessing changes in tumor burden may be useful tools in interpreting the response to immunotherapy.

## Figures and Tables

**Table 1:** Comparison between Lugano lymphoma classification, LYRIC and RECIL 2017.

	Lugano	LYRIC	RECIL 2017
Number of target lesions	Up to 6	Up to 6	Up to 3
Measurement method	Bidimensional: perpendicular diameters	Bidimensional: perpendicular diameters	Unidimensional: long diameter of any target lesion
Complete response (CR)	PET negativity (DS 1–3) with or without a residual mass	Same as Lugano	PET negativity (DS 1–3) plus reduction in SLD > 30%
Minor response (MR)	No	Same as Lugano	Yes: reduction in SLD between ≥10% and <30%
Partial response (PR)	Reduced FDG-PET uptake (DS 4–5) Decrease SPD ≥ 50%	Same as Lugano	Reduction in SLD ≥ 30% not meeting criteria for CR New lesions are included in overall TB
Stable disease (SD)	Stable FDG-PET uptake (DS 4–5) Decrease SPD < 50%	Same as Lugano	Decrease <10% to increase ≤20% in SLD
Progression of disease (PD)	Increased FDG-PET uptake (DS 4–5) Increase SPD ≥ 50% New lesions	As with Lugano, with the exception of IR IR(1): ≥50% increase in SPD in first 12 weeks IR(2): <50% increase in SPD with new lesion(s), or ≥50% increase in PPD of a lesion or set of lesions IR(3): increase in FDG uptake without a concomitant increase in lesion size	Increase in SLD by 20%. For relapse from CR, at least one lesion should measure 2 cm in the long axis with or without PET activity

SPD: sum of product of perpendicular diameters of target lesions. SLD: sum of the longest diameters of target lesions. IR: indeterminate response.

**Table 2:** Contribution of MTV to assessment of response to immunotherapy.

Study	Patients	Treatment	Time Points	Response Criteria	SUV Metrics	Results
Savas et al. (2018) [50]	13 I-IV HL Newly diagnosed	PEM	3 cycles	Lugano	MTV	Mean ΔMTV-90% CR 50%
Dercle et al. (2018) [48]	16 R/R HL	Nivolumab/ PEM	3 mth	Lugano LYRIC	SUVmax SUVmean MTV TLG	ΔMTV-90% in responders (PR+CR) ΔMTV < 50% in refractory (SD+PD)
Castello et al. (2019) [49]	43 R/R HL	Nivolumab/ PEM	Early (8 wks) Interim (17 wks)	Lugano LYRIC	SUVmax SUVmean MTV TLG	ΔMTV > 50% in 29/31 responders (PR+CR) Median MTV-95% CR 60%
Voltin et al. (2020) [51]	53 IIb HL therapy naïve	Nivolumab	4 cycles (16 wks)	Lugano	MTV TLG	Average ΔMTV-91% CR 46.4%

R/R: relapsed/refractory. PEM: pembrolizumab.
